# Protein intake in infancy and kidney size and function at the age of 6 years: The Generation R Study

**DOI:** 10.1007/s00467-015-3096-4

**Published:** 2015-05-09

**Authors:** Trudy Voortman, Hanneke Bakker, Sanaz Sedaghat, Jessica C. Kiefte–de Jong, Albert Hofman, Vincent W. V. Jaddoe, Oscar H. Franco, Edith H. van den Hooven

**Affiliations:** The Generation R Study Group, Erasmus MC, University Medical Center, Rotterdam, The Netherlands; Department of Epidemiology, Erasmus MC, University Medical Center, Rotterdam, The Netherlands; Department of Pediatrics, Erasmus MC, University Medical Center, Rotterdam, The Netherlands; Department of Epidemiology, Erasmus MC, Office Na-2909, PO Box 2040, 3000 Rotterdam, The Netherlands

**Keywords:** Dietary protein, Diet, Children, Kidney volume, Kidney function, Kidney development, Epidemiology

## Abstract

**Background:**

High protein intake has been linked to kidney growth and function. Whether protein intake is related to kidney outcomes in healthy children is unclear.

**Methods:**

We examined the associations between protein intake in infancy and kidney outcomes at age 6 years in 2968 children participating in a population-based cohort study. Protein intake at 1 year was assessed using a food-frequency questionnaire and was adjusted for energy intake. At age 6 years we measured the kidney volume and urinary albumin/creatinine ratio (ACR) of all participating children, and we estimated glomerular filtration rate (eGFR) using serum creatinine and cystatin C levels.

**Results:**

In models adjusted for age, sex, body surface area, and sociodemographic factors, a higher protein intake was associated with a lower ACR and a higher eGFR but was not consistently associated with kidney volume. However, after further adjustment for additional dietary and lifestyle factors, such as sodium intake, diet quality, and television watching, higher protein intake was no longer associated with kidney function. No differences in associations were observed between animal and vegetable protein intake.

**Conclusions:**

Our findings show that protein intake in early childhood is not independently associated with kidney size or function at the age of 6 years. Further study is needed on other early life predictors of kidney size and function in later life.

**Electronic supplementary material:**

The online version of this article (doi:10.1007/s00467-015-3096-4) contains supplementary material, which is available to authorized users.

## Introduction

Kidney function has been shown to track from childhood into adulthood [1]. Subclinical variations in kidney function are already present in childhood and have been found to relate to kidney disease in later life [2], implying that it is important to study determinants of kidney function already in childhood. We have recently observed that reduced infant weight growth is associated with smaller kidney volume in childhood [3] and that longer breastfeeding duration is associated with larger kidney volume and an increased estimated glomerular filtration rate (eGFR) [4]. These observations suggest that exposures in infancy are important for later kidney development.

Dietary protein intake during infancy is a key factor for growth and development and may be associated to kidney growth and function [5]. In animal studies, increased protein intake leads to increased kidney growth and function [6–8], and early postnatal dietary protein affects kidney function [8, 9]. A higher protein intake has also been associated with increased GFR in healthy adults [10–12]. In patients with chronic kidney disease, high protein intake may further reduce kidney function because the kidneys can no longer handle the excretion of protein metabolites [13–16]. However, randomized controlled trials with low-protein diets in adults or children with renal disease have not consistently been able to slow the progression of kidney disease [17–19].

Little is known of the effects of protein intake on kidney function in children with a normal kidney function. Data from trials suggest that infants who received additional dietary protein have larger kidneys [5] and a higher eGFR [20] in infancy than those who received no additional protein. However, whether protein intake in infancy is associated with kidney size and function in later childhood is unknown. Therefore, we examined the associations between protein intake at the age of 1 year and kidney outcomes at the age of 6 years in 2968 children participating in a population-based prospective cohort study. Kidney measures included combined kidney volume, creatinine-based eGFR (eGFR_Creat_), cystatin C-based eGFR (eGFR_CysC_), and urinary albumin/creatinine ratio. We also examined the association between protein intake at age 2 years with kidney outcomes at age 6 years in a subgroup of the children. Additionally, we evaluated whether the associations between protein intake and kidney health differed by protein source, child sex, birth weight, gestational age at birth, kidney volume, or ethnicity.

## Methods

### Study design and population

**Fig. 1:**
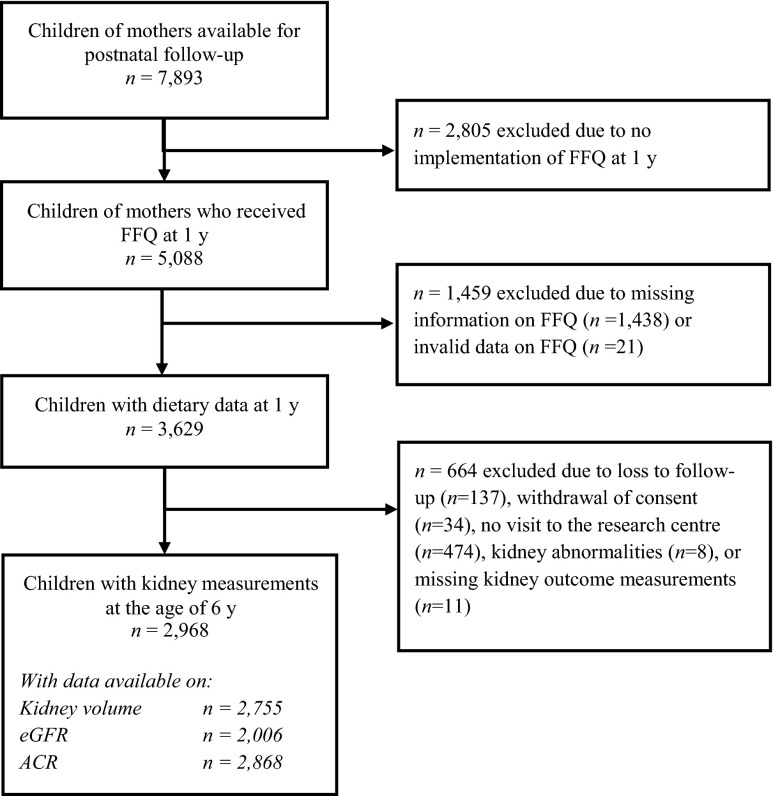
Flow chart of study participants included in the main analysis. *ACR* Albumin/creatinine ratio*, eGFR* estimated glomerular filtration rate*, FFQ* food frequency questionnaire

This study was embedded in the Generation R Study, a population-based prospective cohort study from fetal life onward in Rotterdam, the Netherlands [21]. All children were born between April 2002 and January 2006. The study was conducted according to the guidelines of the Helsinki Declaration and approved by the Medical Ethics Committee of Erasmus Medical Center, Rotterdam (MEC 198.782/2001/31). Written informed consent was given by all parents. A total of 7893 children were available for follow-up studies in early childhood [21]. A questionnaire on child diet around the age of 1 year was sent to 5088 mothers who provided consent for follow-up and had sufficient mastery of the Dutch language (Fig. 1). In total, 3650 (72 %) of these mothers returned the questionnaire [22]. After exclusion of subjects with invalid dietary data and withdrawn consent, information on infant diet was available for 3629 children. From these 3629 children, we excluded children with congenital kidney abnormalities or an albumin–creatinine ratio (ACR) of >25 mg/mmol (*n* = 8) [23]. Of the remaining children, 2968 had one or more kidney measurements available at the age of 6 years (Fig. 1).

### Dietary assessment

Dietary intake was assessed at a median age of 12.9 (95 % range 12.2–18.9) months using a semi-quantitative 211-item food frequency questionnaire (FFQ), as described previously in detail [22, 24]. The FFQ was validated against three 24-h recalls in a representative sample of 32 Dutch children around the age of 1 year living in Rotterdam. The intra-class correlation coefficient was 0.7 for total protein intake [22]. Mothers of a subgroup of 899 Dutch children received an additional FFQ at their child’s median age of 24.9 (95 % range 24.3–27.6) months [24]. Of these children, 715 had kidney measures at the age of 6 years available for analysis [Electronic Supplementary Material (ESM) Fig. S1].

### Kidney outcome assessments

The kidney outcomes of all children were assessed at a median age of 5.9 (95 % range 5.6–6.6) years in a dedicated research center in the Sophia Children’s Hospital in Rotterdam by well-trained staff [23]. Kidney volume was measured with ultrasound, using an ATL-Philips HDI 5000 instrument (Philips Medical Systems, Seattle, WA), equipped with a 2.0- to 5.0-MHz curved array transducer, as described previously in detail [25, 23]. Kidney volume was calculated using the equation for a prolate ellipsoid: volume (cm^3^) = 0.523 × length (cm) × width (cm) × depth (cm) [25]. The combined kidney volume was calculated by summing the right and left kidney volume. We previously reported good intra-observer and inter-observer correlation coefficients using this method [26].

Non-fasting blood samples were drawn by antecubital venipuncture. Creatinine concentrations were measured with enzymatic methods, and cystatin C levels were measured with a particle-enhanced immunoturbidimetric assay (using Cobas 8000 analyzers; Roche, Almere, the Netherlands). Quality control samples demonstrated intra-assay coefficients of variation (CV) of 0.51 and 1.65 % for creatinine and cystatin C, respectively, and inter-assay CV of 1.37 and 1.13 %, respectively [23]. The eGFR_Creat_ was calculated according to the revised Schwartz 2009 formula, which is the most common pediatric equation: eGFR_Creat_ = 36.5 × [height (cm)/ creatinine (μmol/L)] [27]. We also evaluated the eGFR calculated using a cystatin C-based and a combined creatinine and cystatin C formula, as proposed by Zappitelli in 2006: eGFR_CysC_ = 75.94/[cystatin C (mg/L)^1.17^] and eGFR_Combined_ = 507.76 × e^0.003 × height (cm)^/[cystatin C (mg/L)^0.635^ × creatinine (μmol/L)^0.547^] [28].

Urinary creatinine (mmol/L) and albumin (mg/L) levels were measured with an AU analyzer (Beckman Coulter, Brea, CA), and creatinine levels were determined using the Jaffe reaction. The urinary ACR was also calculated. In addition to the continuous ACR, we defined microalbuminuria as an ACR of ≥2.5 mg/mmol for boys and ≥3.5 mg/mmol for girls [29].

### Covariates

Information on maternal age, educational level, and folic acid supplement use was obtained with a questionnaire at enrollment in the study. Maternal height and weight were measured at the research center at enrolment, and body mass index (BMI, kg/m^2^) was calculated. Maternal smoking during pregnancy was assessed using questionnaires in each trimester and was categorized as (1) never; (2) until pregnancy was known; (3) continued during pregnancy. Information on each child’s sex, birth weight and gestational age was available from medical records and hospital registries. Sex- and gestational age-specific standard deviation (SD) scores for birth weight were calculated using Swedish reference data [30]. Child’s ethnicity was defined according to Statistics Netherlands [31] and classified into eight categories (Western, Cape Verdean, Moroccan, Netherlands Antillean, Turkish, Surinamese Creole, Surinamese Hindustani, and other non-Western).

Information on breastfeeding was obtained from delivery reports and postnatal questionnaires, and breastfeeding was categorized as (1) never; (2) partial in the first 4 months; (3) exclusively in the first 4 months of life [22]. Total energy, fat, and sodium intake from foods were estimated using the previously mentioned FFQs and were adjusted for energy intake using the residual method [32]. A previously defined diet score was used to quantify overall diet quality using data obtained with the FFQ [24]. Information on each child’s television watching at around the age of 2 years was obtained using a questionnaire. At the child’s age of 6 years, we measured height and weight at the research center and calculated BMI (kg/m^2^) and body surface area (BSA) using the Du Bois formula: BSA (cm^2^) = weight (kg)^0.425^ × height (cm)^0.725^× 0.007184 [33]. Lean body mass was measured using whole-body dual-energy X-ray absorptiometry scans (General Electric-Lunar, Madison, WI).

### Statistical analysis

We were interested in the effect of protein independent of its energy content and therefore adjusted protein intake for total energy intake using the residual method [32]. Briefly, we used the residuals of a linear regression model with energy intake as the independent variable and protein intake as the dependent variable. These residuals provide a measure of protein intake uncorrelated with total energy intake. To enhance interpretability, predicted protein intake for the mean energy intake (1311 kcal/day) was added to the residuals as a constant [32]. In line with recommendations for dietary exposures, protein intake was analyzed both as a continuous and as a categorical variable [32]. For the latter purpose we categorized protein intake into tertiles and used the lowest tertile as the reference category.

We used multivariable linear regression models to assess the associations of protein intake with combined kidney volume, eGFR, and ACR. We natural log-transformed ACR to obtain a normal distribution. For clinical interpretation, we also assessed the associations of protein intake with the risk of microalbuminuria, using multivariable logistic regression models. Model 1 was adjusted for child’s sex, age, and BSA at kidney measurement. Model 2 was further controlled for specific prenatal and sociodemographic factors, namely, maternal age, maternal educational level, BMI, smoking during pregnancy, and folic acid supplement use, and for child’s ethnicity and birth weight *Z*-score. The final model was additionally adjusted for childhood lifestyle factors: breastfeeding, children’s television watching, total energy, fat and sodium intake, and diet quality score (model 3). Covariates were included in the regression models based on previously shown associations with kidney outcomes [23, 34, 4] or on a significant change (>5 %) in effect estimates. Because both protein intake and kidney volume are strongly related to body size [35] and because creatinine levels are associated with muscle mass [36], we adjusted all models for BSA, and we performed sensitivity analyses in which we replaced BSA by height and weight, by BMI, or by lean body mass. In addition, we examined the association between protein intake and the ratio of kidney volume with either body weight, BMI, or BSA.

To assess whether the associations were different by sex, ethnicity, birth weight, gestational age at birth, or kidney volume at 6 years, we evaluated statistical interactions by adding the product term of the covariate and protein intake to model 2. Stratified analyses were conducted in case the interaction term was significant (*P* < 0.05). Because the FFQ was developed and validated for Dutch children, we performed a sensitivity analysis in Dutch children only. Furthermore, since kidney size and function are different in low birth weight children [25], we performed a sensitivity analysis in children born with a normal birth weight (≥2500 g) and among children born at term (≥37 weeks).

Missing values of covariates were multiple imputed (*n* = 5 imputations) according to the Fully Conditional Specification method (predictive mean matching), assuming no monotone missing pattern [37]. Results are presented as pooled effect estimates after the multiple imputation procedure. Statistical analyses were performed using SPSS version 21.0 (SPSS Inc., Chicago, IL).

## Results

### Subject characteristics

Characteristics of the children and their mothers, stratified by tertiles of protein intake, are presented in Table 1. Mean (±SD) total protein intake at the age of 1 year was 41.2 (±12.9) g, corresponding to 12.9 % of total energy intake (E%). This is higher than recommended for this age group [38], but similar to intakes observed in the general Dutch and other Western pediatric populations [39, 40]. Children in the highest tertile of protein intake had more often been breastfed and were slightly heavier at the age of 6 years. Protein intake at the age of 2 years (13.9 E%) was slightly higher than intake at the age of 1 years (ESM Table S1). At the age of 6 years, mean combined kidney volume was 121 (±21) cm^3^ and mean eGFR_Creat_ was 119 (±16) mL/min per 1.73 m^2^; mean eGFR_CysC_ was lower at 102 (±13) mL/min per 1.73 m^2^. Many children (34 %) had urine albumin levels at or below the detection limit (≤2 mg/L), and microalbuminuria was present in 7.1 % of the children.

**Table 1:** Characteristics of the children and their mothers

Characteristics	All (*n* = 2968)	Tertiles of energy-adjusted total protein intake at age 1 year			*P* value^a^
		Tertile 1 (<37.5 g/day) (*n* = 989)	Tertile 2 (37.5–43.9 g/day) (*n* = 990)	Tertile 3 (>43.9 g/day) (*n* = 989)	
Maternal characteristics					
Maternal age (year)	31.5 (21.7–39.9)	31.8 (22.5–41.4)	31.9 (22.4–39.6)	31.6 (20.6–39.4)	0.01
Maternal body mass index at enrolment (kg/m^2^)	23.4 (18.7–35.2)	23.4 (18.8–34.7)	23.6 (18.9–35.6)	24.5 (18.5–37.1)	0.50
Nulliparous (%)	60.4	59.5	59.8	61.6	0.09
Education level (%)					0.42
Primary	3.5	3.7	3.5	3.2	
Secondary	33.9	33.2	32.8	35.6	
Higher	62.7	63.1	63.7	61.2	
Folic acid supplement use (%)					0.74
Never	15.8	17.0	14.8	15.4	
In the first 10 weeks of pregnancy	30.2	28.5	30.9	31.3	
Periconceptional	54.0	54.4	54.3	53.2	
Smoking during pregnancy (%)					0.49
Never	78.1	78.7	78.6	77.0	
Until pregnancy was known	10.0	9.2	9.4	11.5	
Continued	11.8	12.1	11.9	11.5	
Child characteristics					
Girls (%)	50.9	51.6	52.6	48.5	0.17
Ethnicity (%)					0.24
Western	76.9	75.5	78.1	77.0	
Cape Verdean	2.0	1.8	1.8	2.3	
Moroccan	3.2	2.8	2.8	4.1	
Netherlands Antillean	1.7	2.3	1.9	1.1	
Turkish	4.5	4.5	4.0	4.9	
Surinamese Creoles	2.3	3.0	2.5	1.3	
Surinamese Hindustani	2.2	2.6	1.9	2.2	
Other non-western	7.1	7.5	6.9	7.0	
Gestational age at birth (weeks)	40.0 (1.7)	39.9 (1.9)	39.9 (1.7)	40.0 (1.6)	0.79
Birth weight (g)	3472 (551)	3462 (562)	3466 (557)	3489 (532)	0.51
Breastfeeding (%)					<0.01
Exclusive ≥ 4 months	29.5	34.8	26.9	27.2	
Partial ≥ 4 months	62.5	58.1	65.5	63.7	
Never or ≤ 4 months	8.0	7.1	7.5	9.1	
Child characteristics at dietary measurement					
Age at FFQ (months)	12.9 (12.2–18.9)	12.8 (12.2–18.6)	12.9 (12.2–18.7)	13.0 (12.2–19.4)	<0.01
Total energy intake (kcal/ day)	1266 (678–2212)	1297 (619–2264)	1238 (650–2093)	1253 (765–2237)	0.02
Protein intake (g/day)^b^					
Total protein	41.2 (12.9)	34.9 (10.8)	39.7 (10.1)	48.0 (12.1)	<0.01
Animal protein	25.7 (10.3)	20.8 (8.7)	25.0 (8.1)	33.1 (9.5)	<0.01
Vegetable protein	14.9 (5.7)	13.5 (5.4)	14.8 (5.2)	16.6 (5.9)	<0.01
Protein intake (E%)					
Total protein	12.9 (2.4)	10.5 (1.1)	12.9 (1.0)	15.4 (1.7)	<0.01
Animal protein	8.1 (2.4)	6.2 (1.7)	8.0 (1.6)	10.2 (2.1)	<0.01
Vegetable protein	4.6 (1.4)	4.1 (1.3)	4.7 (1.3)	5.1 (1.3)	<0.01
Total fat intake (g/day)^b^	42.3 (17.5)	43.1 (18.8)	40.8 (16.0)	42.7 (16.7)	<0.01
Sodium intake from foods (g/day)^b^	1.02 (0.35)	0.88 (0.32)	0.98 (0.30)	1.17 (0.35)	<0.01
Television watching (h/day)	0.9 (0.5)	0.9 (0.5)	0.9 (0.5)	0.9 (0.5)	0.19
Diet score	4.2 (1.3)	3.3 (1.1)	4.1 (1.1)	5.1 (1.2)	<0.01
Child characteristics at 6-year visit					
Age (years)	5.9 (5.6–6.6)	5.9 (5.6–6.5)	5.9 (5.6–6.6)	5.9 (5.6–6.6)	0.03
Height (cm)	118.2 (5.2)	117.8 (4.9)	118.2 (5.4)	118.5 (5.2)	0.02
Weight (kg)	22.4 (3.4)	22.1 (3.1)	22.4 (3.6)	22.7 (3.5)	<0.01
Body mass index (kg/m^2^)	16.0 (1.6)	15.9 (1.5)	16.0 (1.6)	16.1 (1.7)	<0.01
Body surface area (kg/m^2^)	0.86 (0.08)	0.85 (0.07)	0.86 (0.08)	0.86 (0.08)	<0.01
Combined kidney volume (cm^3^)	121 (21)	119 (21)	122 (23)	122 (21)	<0.01
Creatinine (μmol/l)	37.0 (5.2)	37.2 (5.0)	36.8 (5.4)	36.8 (5.2)	0.32
Cystatin C (mg/L)	0.79 (0.08)	0.79 (0.08)	0.78 (0.08)	0.78 (0.08)	0.02
eGFR_Creat_ (Schwartz) (mL/min per 1.73 m^2^)	119 (16)	118 (15)	120 (17)	120 (16)	0.06
eGFR_CysC_ (Zappitelli) (mL/min per 1.73 m^2^)	102 (13)	100 (13)	102 (14)	102 (13)	<0.01
Urinary albumin/creatinine ratio	0.79 (0.20–5.70)	0.83 (0.20–7.33)	0.77 (0.190–5.56)	0.77 (0.20–5.00)	0.01
Microalbuminuria (%)	7.1	7.6	7.5	6.3	0.49

Values are given as percentages for categorical variables, as means with the standard deviation (SD) in parenthesis for continuous variables with a normal distribution, or as medians with the 95% range in parenthesis for continuous variables with a skewed distribution

E%, Energy percentage; eGFR, estimated glomerular filtration rate; FFQ, food frequency questionnaire

^a^ *P* values for differences of means between the tertiles of protein intake, assessed using analysis of variance for continuous variables with a normal distribution, Kruskal-Wallis test for continuous variables with a skewed distribution, and chi-square tests for categorical variables

^b^Not adjusted for energy intake

### Protein intake in early childhood and kidney outcomes at school age

Table 2 presents the associations between protein intake and kidney outcomes. In model 1, adjusted for age, sex, and BSA, a higher protein intake at the age of 1 year was associated with a higher eGFR and lower ACR at the age of 6 years. Protein intake was not consistently associated with kidney volume. Results for eGFR_CysC_ were similar to those for eGFR_Creat_ (Table 2) and for eGFR_Combined_ (ESM Table S2). After further adjustment for sociodemographic variables and maternal factors (model 2), the effect estimates hardly changed and a higher protein intake remained significantly associated with a higher eGFR and a lower ACR. However, after further adjustment for child lifestyle factor (model 3), all associations attenuated towards null (Table 2). Important lifestyle confounders in the associations with kidney outcomes were child’s television watching, overall diet quality, and sodium intake.

**Table 2:** Associations of protein intake at age 1 year with childhood kidney volume and function at age 6 years (*n* = 2968)

Protein intake	Kidney volume (mm^3^) (*n* = 2755)	eGFR_Creat_ (Schwartz 2009) (mL/min per 1.73 m^2^) (*n* = 2006)	eGFR_CysC_ (Zappitelli 2006) (mL/min per 1.73 m^2^) (*n* = 2007)	ACR (% change)^a^ (*n* = 2868)
Model 1^b^				
Tertile 1	Reference	Reference	Reference	Reference
Tertile 2	2.31 (0.61, 4.02)*	1.90 (0.17, 3.63)*	1.84 (0.44, 3.25)*	−6.8 (−14.6, 1.0)
Tertile 3	1.16 (−0.54, 2.87)	2.46 (0.73, 4.19)*	1.75 (0.35, 3.15)*	−7.8 (−15.7, −0.1)*
* P*_trend_^c^	0.17	<0.01*	0.01*	0.04*
Per 10 g	0.29 (−0.67, 1.25)	1.03 (0.04, 1.99)*	0.66 (−0.12, 1.44)	−5.4 (−9.8, −1.1)*
Model 2^b^				
Tertile 1	Reference	Reference	Reference	Reference
Tertile 2	2.33 (0.64, 4.03)*	1.85 (0.12, 3.58)*	1.84 (0.43, 3.25)*	−6.7 (−14.5, 1.1)
Tertile 3	1.21 (−0.50, 2.91)	2.28 (0.56, 4.00)*	1.70 (0.30, 3.11)*	−6.9 (−14.8, 0.0)*
* P*_trend_^c^	0.16	<0.01*	0.02*	0.08
Per 10 g	0.31 (−0.65, 1.27)	0.91 (−0.05, 1.86)	0.64 (−0.14, 1.43)	−4.9 (−9.3, −0.01)*
Model 3^b^				
Tertile 1	Reference	Reference	Reference	Reference
Tertile 2	1.96 (0.10, 3.82)*	1.21 (−0.69, 3.11)	1.58 (−0.21, 3.33)	−3.1 (−11.7, 5.4)
Tertile 3	0.36 (−1.91, 2.63)	1.11 (−1.20, 3.42)	1.60 (−0.28, 3.49)	−0.4 (−10.9, 10.0)
* P*_trend_^c^	0.74	0.35	0.10	0.93
Per 10 g	−0.55 (−1.86, 0.76)	−0.17 (−1.49, 1.15)	0.37 (−0.71, 1.45)	−2.0 (−8.0, 4.0)

*Significant at *P* < 0.05

Values are based on multivariable linear regression models and reflect differences or percentage change (95 % confidence interval given in parenthesis) in kidney outcomes for tertiles of protein intake compared to the lowest tertile, and per 10 g of protein intake per day

^a^The albumin/creatinine ratio (ACR) is log-transformed, therefore the regression coefficients reflect the percentage change rather than the absolute difference

^b^Protein intake is energy-adjusted using the nutrient residual method: Model 1 is adjusted for child’s sex, age, and body surface area at the 6-year visit; Model 2 is additionally adjusted for maternal age, educational level, and body mass index at enrolment, for smoking and folic acid supplement use during pregnancy, and for children’s ethnicity and gestational-age adjusted birth weight; Model 3 is additionally adjusted for breastfeeding in the first 4 months of life, children’s television watching, total energy intake, energy-adjusted total fat intake, energy-adjusted sodium intake, and diet quality score

^c^ *P*_trend_ is obtained by including the number of the tertiles of protein intake as continuous variable in the model

### Additional analyses

No clear differences were observed for the associations of animal versus vegetable protein intake on kidney outcomes (ESM Table S3). Replacement of BSA with either height and weight, BMI, or lean body mass, or the replacement of absolute kidney volume by the ratio of kidney volume with weight, BMI, or BSA did not change the effect estimates (data not shown). Protein intake was not associated with urinary albumin or creatinine levels (data not shown). No significant interactions were observed between total protein intake and sex, birth weight, gestational age, ethnicity, or kidney volume on any of the kidney outcomes. Sensitivity analyses in Dutch children only (*n* = 1994) showed similar patterns of associations as in the whole group, but with slightly larger effect estimates and smaller *P* values (ESM Table S4). Among children born with a normal birth weight (≥2500 g, *n* = 2802) and among children born at term (≥37 weeks, *n* = 2781), effect estimates were similar to those observed in the whole group (data not shown). In line with the results for protein intake at the age of 1 year, higher total protein intake at the age of 2 years was associated with a higher eGFR_Creat_ and a trend towards a lower ACR in crude models, and the associations attenuated to null after adjustment for other lifestyle factors (ESM Table S5). In contrast to protein intake at the age of 1 year, protein intake at 2 years was not associated with eGFR_cysC_ in crude models and was associated with a higher kidney volume, which was explained by other lifestyle factors (ESM Table S5).

## Discussion

We have examined the associations between protein intake in early childhood and kidney size and function at school age in a large prospective population-based cohort study. We observed that associations between higher protein intake in infancy and higher eGFR and lower ACR at the age of 6 years were explained by other dietary and lifestyle factors of the children, such as sodium intake and television watching. Furthermore, protein intake was not associated with kidney size, and no differences in associations were observed for animal versus vegetable protein intake.

### Interpretation and comparison with previous studies

Contrary to findings of previous studies in infants and adults, protein intake in infancy was not consistently associated with combined kidney volume in our population-based sample of school-age children. In a multi-center trial in several European countries, healthy infants who received the higher protein infant formula had higher kidney volumes at the age of 6 months than infants receiving the lower protein formula [5]. Whether this difference in kidney volume persisted until later age was not studied. A previous observational study in 631 healthy infants in Denmark reported that 3-month-old infants who received formula feeding had a larger kidney size than infants who received breastfeeding, and the authors hypothesized that the effect might be attributable to the higher protein content in infant formula [41]. However, the difference was no longer present at 18 months of age [41]. In line with this, in a study in young rats that received isocaloric high or low protein diets after weaning, a higher protein intake increased kidney size [6]. However, 1 month after discontinuation of the high protein diet, kidney size was comparable to that of the rats fed low protein diets. These studies suggest that the effect of protein intake on kidney growth could be reversible. In our study, kidney outcomes were measured a few years after the assessment of dietary protein intake. Therefore, we could speculate that a potential effect of protein intake in early life on kidney size may no longer be apparent in the children at the age of 6 years. Kidney hypertrophy in response to high protein intake could be a compensatory response to higher levels of nitrogenous protein metabolites (such as urea) and may be temporary response [15]. Alternatively, hypertrophy of the kidney in response to protein intake may occur via increased insulin-like growth factor-1 secretion, which may lead to permanent changes in kidney size [42, 43].

We observed a higher eGFR in relation to higher protein intake, but this association was explained by other lifestyle factors. Important confounding factors were television watching, overall diet quality, and breastfeeding in early infancy. This result is in contrast to the finding of a higher eGFR with additional dietary protein in a small trial in preterm born infants [20] and in short-term trials in adults [12, 44, 11]. However, in line with our results, the previously mentioned large trial in healthy infants did not report an effect of a higher protein infant formula on eGFR at the age of 6 months [5], and an observational study in healthy infants reported no associations between intake of infant formula and eGFR [41]. In a previous study also embedded in the Generation R Study, higher maternal protein intake during pregnancy was associated with higher eGFR of the child, independent of protein intake in infancy (reference: doi: 10.3945/ajcn.114.102228). Similar to kidney growth, a higher GFR in response to high protein intake is considered to be an adaptive responses to high levels of circulating protein metabolites which will increase the workload of the kidneys and may lead to hyperfiltration [15]. Similar to the effect on kidney growth, this response may be reversible [6].

In our population, a higher protein intake was associated with a lower ACR in crude models, but this was no longer significant after adjustment for other dietary factors, such as sodium intake. This observation is in contrast to results from a trial in healthy adults which showed that increased protein intake increases urinary albumin levels [12]. However, in line with our results, a few other trials have reported the absence of any association between protein intake and urinary albumin excretion [11, 44].

In contrast to previous observational studies in adults [45–47], we did not observe clear differences in associations for animal and vegetable protein intake, and in contrast to studies in animals [7, 43], we also did not observe significant interactions between sex of the child and protein intake on kidney health. We also did not observe any interaction between protein intake and birth weight or gestational age at birth. The results of our study do not indicate that changes in dietary recommendations for healthy infants are required with respect to later kidney health. However, further studies are needed to assess whether protein intake may specifically affect kidney outcomes in preterm or small-for-gestational age born children.

### Strengths and limitations

An important strength of our study is its prospective design within a large population-based cohort. We had information available on protein intake and kidney outcomes for almost 3000 children, and we had information on many potential maternal and child confounders which have not always been considered in previous observational studies.

A limitation of our dietary assessment methods is that an FFQ relies on memory, and reported food intakes are subject to measurement error [48]. However, validation of our FFQ against three 24-h recalls showed a good intra-class correlation coefficient for protein intake. Another limitation of our FFQ is that it has only been validated for Dutch children [22, 24]. However, sensitivity analyses in Dutch children only showed similar results. The strengths of our dietary assessment are that an FFQ measures habitual diet rather than dietary intake on just 1 or a few days and that we calculated not only total protein, but also protein from animal and vegetable sources separately. A limitation of our study is that dietary data for the children at age 6 years were not available; consequently, we were unable to assess the association of current diet with kidney health.

We performed detailed kidney measurements using ultrasound to measure kidney volume, and we had blood and urine samples to estimate kidney function. Unfortunately, we did not measure inulin clearance to calculate actual GFR. To estimate GFR we used a creatinine-based formula that has been validated and which is widely used in pediatric populations [27]. A limitation of serum creatinine as a marker of kidney function is its strong relationship with muscle mass [36]. Cystatin C is a more sensitive marker for kidney function in pediatric populations than serum creatinine since it is not affected by child age, height, or weight [49, 50], and we therefore also evaluated eGFR based on cystatin C levels [28]. This latter formula has been evaluated against inulin clearance and compared with other eGFR formulas and found to be accurate and precise in estimating GFR in addition to the Schwartz 2009 formula [36].

### Conclusions

In this prospective cohort study, associations between protein intake in early childhood and kidney function at the age of 6 years were explained by other dietary and lifestyle factors of the children. Furthermore, protein intake was not associated with kidney size, and no differences in associations were observed for animal versus vegetable protein intake.

## Electronic supplementary material

ESM 1 (DOCX 57 kb)

## References

[1] Singh A, Satchell SC (2011). Microalbuminuria: causes and implications. Pediatr Nephrol.

[2] Luyckx VA, Bertram JF, Brenner BM, Fall C, Hoy WE, Ozanne SE, Vikse BE (2013). Effect of fetal and child health on kidney development and long-term risk of hypertension and kidney disease. Lancet.

[3] Bakker H, Gaillard R, Franco OH, Hofman A, van der Heijden AJ, Steegers EAP, Taal HR, Jaddoe VWV (2014). Fetal and infant growth patterns and kidney function at school age. J Am Soc Nephrol.

[4] Miliku K, Voortman T, Bakker H, Hofman A, Franco O, Jaddoe V (2015). Infant breastfeeding and kidney function in school-aged children. Am J Kidney Dis.

[5] Escribano J, Luque V, Ferre N, Zaragoza-Jordana M, Grote V, Koletzko B, Gruszfeld D, Socha P, Dain E, Van Hees JN, Verduci E, Closa-Monasterolo R (2011). Increased protein intake augments kidney volume and function in healthy infants. Kidney Int.

[6] Jakobsson B, Celsi G, Lindblad BS, Aperia A (1987). Influence of different protein intake on renal growth in young rats. Acta Paediatr Scand.

[7] Hammond KA, Janes DN (1998). The effects of increased protein intake on kidney size and function. J Exp Biol.

[8] Hoppe CC, Evans RG, Moritz KM, Cullen-McEwen LA, Fitzgerald SM, Dowling J, Bertram JF (2007). Combined prenatal and postnatal protein restriction influences adult kidney structure, function, and arterial pressure. Am J Physiol Regul Integr Comp Physiol.

[9] Siddique K, Guzman GL, Gattineni J, Baum M (2014). Effect of postnatal maternal protein intake on prenatal programming of hypertension. Reprod Sci.

[10] Schwingshackl L, Hoffmann G (2014). Comparison of high vs. normal/low protein diets on renal function in subjects without chronic kidney disease: a systematic review and meta-analysis. PLoS One.

[11] Skov AR, Toubro S, Bülow J, Krabbe K, Parving HH, Astrup A (1999). Changes in renal function during weight loss induced by high vs low-protein low-fat diets in overweight subjects. Int J Obes.

[12] Frank H, Graf J, Amann-Gassner U, Bratke R, Daniel H, Heemann U, Hauner H (2009). Effect of short-term high-protein compared with normal-protein diets on renal hemodynamics and associated variables in healthy young men. Am J Clin Nutr.

[13] Kasiske BL, Lakatua JDA, Ma JZ, Louis TA (1998). A meta-analysis of the effects of dietary protein restriction on the rate of decline in renal function. Am J Kidney Dis.

[14] Knight EL, Stampfer MJ, Hankinson SE, Spiegelman D, Curhan GC (2003). The impact of protein intake on renal function decline in women with normal renal function or mild renal insufficiency. Ann Intern Med.

[15] King AJ, Levey AS (1993). Dietary-protein and renal-function. J Am Soc Nephrol.

[16] Friedman AN (2004). High-protein diets: Potential effects on the kidney in renal health and disease. Am J Kidney Dis.

[17] Chaturvedi S, Jones C (2007). Protein restriction for children with chronic renal failure. Cochrane Database Syst Rev.

[18] Fouque D, Laville M (2009). Low protein diets for chronic kidney disease in non diabetic adults (Review). Cochrane Database Syst Rev.

[19] Robertson L, Waugh N, Robertson A (2007). Protein restriction for diabetic renal disease. Cochrane Database Syst Rev.

[20] Herin P, Zetterstrom R (1987). Studies in renal response to various protein intakes in preterm infants. Acta Paediatr Scand.

[21] Jaddoe VWV, van Duijn CM, Franco OH, van der Heijden AJ, van IIzendoorn MH, de Jongste JC, van der Lugt A, Mackenbach JP, Moll HA, Raat H, Rivadeneira F, Steegers EAP, Tiemeier H, Uitterlinden AG, Verhulst FC, Hofman A (2012). The Generation R Study: design and cohort update 2012. Eur J Epidemiol.

[22] Kiefte-de Jong JC, de Vries JH, Bleeker SE, Jaddoe VW, Hofman A, Raat H, Moll HA (2013). Socio-demographic and lifestyle determinants of ‘Western-like’ and ‘Health conscious’ dietary patterns in toddlers. Br J Nutr.

[23] Bakker H, Kooijman MN, van der Heijden AJ, Hofman A, Franco OH, Taal HR, Jaddoe VWV (2014). Kidney size and function in a multi-ethnic population-based cohort of school-age children. Pediatr Nephrol.

[24] Voortman T, Kiefte-de Jong JC, Geelen A, Villamor E, Moll HA, de Jongste JC, Raat H, Hofman A, Jaddoe VWV, Franco OH, van den Hooven EH (2015). The development of a diet quality score for preschool children and its validation and determinants in the Generation R Study. J Nutr.

[25] Geelhoed JJM, Taal HR, Steegers EAP, Arends LR, Lequin M, Moll HA, Hofman A, van der Heijden AJ, Jaddoe VWV (2010). Kidney growth curves in healthy children from the third trimester of pregnancy until the age of two years. The Generation R Study. Pediatr Nephrol.

[26] Geelhoed JJM, Kleyburg-Linkers VE, Snijders SPE, Lequin M, Nauta J, Steegers EAP, van der Heijden AJ, Jaddoe VWV (2009). Reliability of renal ultrasound measurements in children. Pediatr Nephrol.

[27] Schwartz GJ, Munoz A, Schneider MF, Mak RH, Kaskel F, Warady BA, Furth SL (2009). New equations to estimate GFR in children with CKD. J Am Soc Nephrol.

[28] Zappitelli M, Parvex P, Joseph L, Paradis G, Grey V, Lau S, Bell L (2006). Derivation and validation of cystatin C-based prediction equations for GFR in children. Am J Kidney Dis.

[29] Donaghue KC, Chiarelli F, Trotta D, Allgrove J, Dahl-Jorgensen K, International Society for Pediatric Adolescent Diabetes (ISPAD) (2007). ISPAD clinical practice consensus guidelines 2006–2007. Microvascular and macrovascular complications. Pediatr Diabetes.

[30] Niklasson A, Ericson A, Fryer JG, Karlberg J, Lawrence C, Karlberg P (1991). An update of the swedish reference-standards for weight, length and head circumference at birth for given gestational-Age (1977–1981). Acta Paediatr Scand.

[31] Statistics Netherlands (2004) Immigrants in the Netherlands 2004 (Allochtonen in Nederland 2004). Statistics Netherlands (Centraal Bureau voor de Statistiek), Den Haag/Heerlen

[32] Willett WC, Howe GR, Kushi LH (1997). Adjustment for total energy intake in epidemiologic studies. Am J Clin Nutr.

[33] Du Bois D, Du Bois EF (1916). A formula to estimate the approximate surface area if height and weight be known. Arch Int Med.

[34] Kooijman MN, Bakker H, Franco OH, Hofman A, Taal HR, Jaddoe VW (2015). Fetal smoke exposure and kidney outcomes in school-aged children. Am J Kidney Dis.

[35] Schmidt IM, Molgaard C, Main KM, Michaelsen KF (2001). Effect of gender and lean body mass on kidney size in healthy 10-year-old children. Pediatr Nephrol.

[36] Bacchetta J, Cochat P, Rognant N, Ranchin B, Hadj-Aissa A, Dubourg L (2011). Which creatinine and cystatin C equations can be reliably used in children?. Clin J Am Soc Nephrol.

[37] Sterne JA, White IR, Carlin JB, Spratt M, Royston P, Kenward MG, Wood AM, Carpenter JR (2009). Multiple imputation for missing data in epidemiological and clinical research: potential and pitfalls. BMJ.

[38] Institute of Medicine (2002/2005) Dietary reference intakes for energy, carbohydrate. Fiber, fat, fatty acids, cholesterol, protein, and amino acids. National Academy of Sciences, Washington DC 10.1016/s0002-8223(02)90346-9 12449285

[39] Hornell A, Lagstrom H, Lande B, Thorsdottir I (2013). Protein intake from 0 to 18 years of age and its relation to health: a systematic literature review for the 5th Nordic Nutrition Recommendations. Food Nutr Res.

[40] Ocké MC, van Rossum CTM, Fransen HP, Buurma EJM, Boer EJd, Brants HAM, Niekerk EM, Laan JDvd, Drijvers JJMM, Ghameshlou Z (2008) Dutch National Food Consumption Survey—Young children 2005/2006. National Institute for Public Health and the Environment (RIVM), Bilthoven

[41] Schmidt IM, Damgaard IN, Boisen KA, Mau C, Chellakooty M, Olgaard K, Main KM (2004). Increased kidney growth in formula-fed versus breast-fed healthy infants. Pediatr Nephrol.

[42] Luque V, Escribano J, Grote V, Ferre N, Koletzko B, Gruszfeld D, Socha P, Langhendries JP, Goyens P, Closa-Monasterolo R, European Childhood Obesity P (2013). Does insulin-like growth factor-1 mediate protein-induced kidney growth in infants? A secondary analysis from a randomized controlled trial. Pediatr Res.

[43] Murray BM, Brown GP, Schoenl M (1998). Interaction of gender and dietary protein on renal growth and the renal growth hormone-insulin-like growth factor axis. J Lab Clin Med.

[44] Solling K, Christensen CK, Solling J, Christiansen JS, Mogensen CE (1986). Effect on renal haemodynamics, glomerular filtration rate and albumin excretion of high oral protein load. Scand J Clin Lab Invest.

[45] Kontessis P, Jones S, Dodds R, Trevisan R, Nosadini R, Fioretto P, Borsato M, Sacerdoti D, Viberti GC (1990). Renal, metabolic and hormonal responses to ingestion of animal and vegetable proteins. Kidney Int.

[46] Nettleton JA, Steffen LM, Palmas W, Burke GL, Jacobs DR (2008). Associations between microalbuminuria and animal foods, plant foods, and dietary patterns in the multiethnic study of atherosclerosis. Am J Clin Nutr.

[47] Toeller M, Buyken A, Heitkamp G, Bramswig S, Mann J, Milne R, Gries FA, Keen H (1997). Protein intake and urinary albumin excretion rates in the EURODIAB IDDM Complications Study. Diabetologia.

[48] Kipnis V, Subar AF, Midthune D, Freedman LS, Ballard-Barbash R, Troiano RP, Bingham S, Schoeller DA, Schatzkin A, Carroll RJ (2003). Structure of dietary measurement error: results of the OPEN biomarker study. Am J Epidemiol.

[49] Shlipak MG, Coresh J, Gansevoort RT (2013). Cystatin C versus creatinine for kidney function-based risk. New Engl J Med.

[50] Finney H, Newman DJ, Thakkar H, Fell JME, Price CP (2000). Reference ranges for plasma cystatin C and creatinine measurements in premature infants, neonates, and older children. Arch Dis Child.

