# Supplementary material for: Protein intake in infancy and kidney size and function at the age of 6 years: The Generation R Study
Source: Pediatr Nephrol. 2015 May 9;30(10):1825–33. doi: 10.1007/s00467-015-3096-4 (PMC4549379; doi:10.1007/s00467-015-3096-4)
Supplement: Supplementary file 1 — (DOCX 57 kb) [file 467_2015_3096_MOESM1_ESM.docx]

Supplementary Material

Protein intake in infancy and kidney size and function at the age of 6 years: The Generation R Study

**Supplementary Figure S1.** Population for analysis for children with dietary data at the age of 2 y.

**Supplementary Table S1.** . Dietary characteristics for children with dietary data at 2 y.

**Supplementary Table S2.** Associations of protein intake at 1 y with serum creatinine and cystatin C levels, and microalbuminuria at 6 y.

**Supplementary Table S3.** Associations of animal and vegetable protein intake at 1 y with kidney volume and function at 6 y.

**Supplementary Table S4.** Associations of protein intake at the age of 1 y with kidney volume and function at 6 y in Dutch children.

**Supplementary Table S5.** Associations of protein intake at 2 y with childhood kidney volume and function at 6 y.

Supplementary Figure S1. Population for analysis for children with dietary data at the age of 2 y.

Children of mothers who received FFQ at 2 y

*n* = 899

Children of mothers participating in focus cohort

*n* = 1,232

*n* = 207 excluded due to no implementation of FFQ at 2 y

*n* = 55 excluded due to missing information on FFQ

Children with dietary data at 2 y

*n* = 844

*n* = 129 excluded due to loss to follow-up (*n* =8), withdrawal of consent (*n* =7), no visit to the research centre (*n* =111), twins (*n* =0), kidney abnormalities (*n* =1), or missing kidney outcome measurements (*n* =2)

Children with kidney measurements at the age of 6 y

*n* = 715

*With data available on:*

*Kidney volume n = 660*

*eGFR n = 474*

*ACR n = 685*

Supplementary Table S1. Dietary characteristics for children with dietary data at 2 y.^a^

|  |  | |  | **Tertiles of energy-adjusted total protein intake at 2 y** | | | | | |  | |  |  |
| --- | --- | --- | --- | --- | --- | --- | --- | --- | --- | --- | --- | --- | --- |
|  | | **All**  **(n=715)** | | | **Tertile 1 (<43.0 g/d)**  **(n=238)** | | **Tertile 2 (43.0-48.2 g/d)**  **(n=239)** | **Tertile 3 (>48.2 g/d)**  **(n=238)** | ***P value ^b^*** | |  |  |  |
| Age at FFQ (mo) | | 24.9 (24.3-27.6) | | | 24.9 (24.3-27.7) | | 24.9 (24.3-27.5) | 24.9 (24.3-27.2) | *0.48* | |  |  |  |
| Total energy intake (kcal/d) | | 1305 (838-1959) | | | 1336 (842-2049) | | 1244 (802-1896) | 1310 (936-1996) | *<0.01* | |  |  |  |
| Protein intake (g/d) ^c^ | | | | | |  | | |  | |  | |  |
| - Total protein | | 46.0 (10.8) | | | 40.0 (9.3) | | 44.3 (8.7) | 52.4 (9.8) | *<0.01* | |  |  |  |
| - Animal protein | | 27.9 (8.8) | | | 22.0 (6.8) | | 26.7 (6.4) | 34.7 (8.0) | *<0.01* | |  |  |  |
| - Vegetable protein | | 18.1 (5.1) | | | 17.7 (5.0) | | 17.6 (5.3) | 18.9 (5.4) | *<0.01* | |  |  |  |
| Total fat intake (g/d) ^c^ | | 46.5 (13.4) | | | 44.7 (14.4) | | 44.4 (12.5) | 50.4 (12.5) | *<0.01* | |  |  |  |
| Sodium intake from foods (g/d) ^c^ | | 1.28 (0.32) | | | 1.12 (0.30) | | 1.21 (0.29) | 1.43 (0.33) | *<0.01* | |  |  |  |
| Protein intake (E%) | | | | | |  | | |  | |  | |  |
| - Total protein | | 13.9 (1.9) | | | 11.8 (1.1) | | 14.0 (0.7) | 15.9 (1.1) | *<0.01* | |  |  |  |
| - Animal protein | | 8.4 (2.1) | | | 6.5 (1.4) | | 8.5 (1.4) | 10.3 (1.7) | *<0.01* | |  |  |  |
| - Vegetable protein | | 5.5 (1.2) | | | 5.1 (1.1) | | 5.5 (1.2) | 5.6 (1.3) | *<0.01* | |  |  |  |

*^a^ Values are means (SD) for continuous variables with a normal distribution or medians (95% range) for continuous variables with a skewed distribution.*

*^b^ p-values for differences of means between the tertiles of protein intake, assessed using ANOVA for continuous variables with a normal distribution and Kruskal-Wallis test for continuous variables with a skewed distribution.*

*^c^ Not adjusted for energy intake*

*Abbreviations: E%, energy percentage, FFQ, food frequency questionnaire.*

Supplementary Table S2. Associations of protein intake at 1 y with serum creatinine and cystatin C levels, eGFR_combined_ and microalbuminuria at 6 y.^a^

|  | **Serum creatinine (µmol/l)** | **Serum cystatin C (µg/l)** | **eGFR_Ccombined_**  **(Zappitelli 2006)**  **(ml/min per 1.73m²)** | **Microalbuminuria**  **(OR)** |
| --- | --- | --- | --- | --- |
| **Protein intake** | **n = 2,006** | **n = 2,007** | **n = 2,007** | **n = 2,868** |
| **Model 1^b^** |  |  |  |  |
| Tertile 1 | *Reference* | *Reference* | *Reference* | *Reference* |
| Tertile 2 | -0.51 (-1.05, 0.04) | **-10.5 (-18.9, -2.2)** | **2.23 (0.76, 3.71)** | 1.11 (0.79, 1.57) |
| Tertile 3 | **-0.81 (-1.35, -0.27)** | **-11.0 (-19.3, -2.7)** | **2.46 (0.99, 3.94)** | 0.86 (0.60, 1.24) |
| *P_trend_ ^c^* | ***<0.01*** | ***<0.01*** | ***<0.01*** | *0.44* |
| Per 10 g | **-0.35 (-0.65, -0.05)** | -3.9 (-8.6, 0.7) | **0.96 (0.14, 1.78)** | 0.91 (0.75, 1.11) |
| **Model 2^b^** |  |  |  |  |
| Tertile 1 | *Reference* | *Reference* | *Reference* | *Reference* |
| Tertile 2 | -0.50 (-1.04, 0.04) | **-10.7 (-20.4, -2.3)** | **2.22 (0.74, 3.70)** | 1.21 (0.85, 1.75) |
| Tertile 3 | **-0.74 (-1.28, -0.20)** | **-10.9 (-19.2, -2.5)** | **2.33 (0.86, 3.80)** | 1.05 (0.8, 1.62) |
| *P_trend_ ^c^* | ***<0.01*** | *0.01* | ***<0.01*** | *0.49* |
| Per 10 g | **-0.31 (-0.61, -0.01)** | -3.9 (-8.6, 0.7) | **0.88 (0.07, 1.70)** | 0.95 (0.75, 1.19) |
| **Model 3^b^** |  |  |  |  |
| Tertile 1 | *Reference* | *Reference* | *Reference* | *Reference* |
| Tertile 2 | -0.27 (-0.86, 0.33) | **-10.5 (-19.7, -1.2)** | 1.61 (-0.25, 3.44) | 1.27 (0.87, 1.85) |
| Tertile 3 | -0.32 (-1.04, 0.40) | -10.5 (-21.7, 0.7) | 1.60 (-0.38, 3.58) | 1.10 (0.69, 1.78) |
| *P_trend_ ^c^* | *0.39* | *0.07* | *0.12* | *0.77* |
| Per 10 g | 0.05 (-0.36, 0.47) | -2.1 (-8.5, 4.3) | 0.08 (-1.06, 1.21) | 1.04 (0.80, 1.36) |

*^a^ Values are based on multivariable linear regression models and reflect differences or percentage change (95%CI) in kidney outcomes for tertiles of protein intake compared to the lowest tertile, and per 10 g of protein intake per day. Bold numbers indicate statistically significant results (P<0.05)*

*^b^Protein intake is energy-adjusted using the nutrient residual method.*

*Model 1 is adjusted for child’s sex, age and body surface area at 6 y visit.*

*Model 2 is additionally adjusted for maternal age, educational level, and BMI at enrolment, for smoking and folic acid supplement use during pregnancy, and for children’s ethnicity, and gestational-age adjusted birth weight.*

*Model 3 is additionally adjusted for breastfeeding in the first four months of life, children’s television watching, total energy intake, energy-adjusted total fat intake, energy-adjusted sodium intake, and diet quality score.*

*^c^ P_trend_ was obtained by including the number of the tertiles of protein intake as continuous variable in the model*

*Abbreviations: eGFR, estimated glomerular filtration rate; OR, odds ratio*

Supplementary Table S3. Associations of animal and vegetable protein intake at 1 y with kidney volume and function at 6 y. ^a^

|  | **Kidney volume**  **(mm3)** | | **eGFR_Creat_**  **(Schwartz 2009)**  **(ml/min/1.73m²)** | | **eGFR_CysC_**  **(Zappitelli 2006)**  **(ml/min/1.73m²)** | | **ACR**  **(% change) ^c^** |
| --- | --- | --- | --- | --- | --- | --- | --- |
|  | **n = 2,755** | | **n = 2,006** | | **n = 2,007** | | **n = 2,868** |
| ***Animal protein intake ^b^*** | |  | | | |  | |
| Tertile 1 | *Reference* | | | *Reference* | *Reference* | | *Reference* |
| Tertile 2 | 1.31 (-0.46, 3.07) | | | -0.43 (-2.21, 1.36) | 0.27 (-1.19, 1.72) | | 3.6 (-4.5, 11.8) |
| Tertile 3 | -0.51 (-2.35, 1.34) | | | 1.11 (-0.75, 2.96) | 0.24 (-1.27, 1.76) | | 4.9 (-3.6, 13.4) |
| *P_trend_ ^d^* | *0.43* | | | *0.27* | *0.29* | | *0.34* |
| Per 10 g | *-0.56 (-1.82, 0.70)* | | | *0.38 (-0.89, 1.65)* | *0.52 (-0.52, 1.56)* | | *-2.0 (-7.7, 3.8)* |
| ***Vegetable protein intake ^b^*** | |  | | | |  | |
| Tertile 1 | *Reference* | | | *Reference* | *Reference* | | *Reference* |
| Tertile 2 | 1.08 (-0.88, 3.03) | | | 0.50 (-1.46, 2.46) | 1.09 (-0.52, 2.70) | | -2.5 (-11.5, 6.5) |
| Tertile 3 | -1.24 (-3.59, 1.10) | | | 0.96 (-1.41, 3.33) | 0.61 (-1.33, 2.55) | | 0.7 (-10.1, 11.6) |
| *P_trend_ ^d^* | *0.25* | | | *0.45* | *0.36* | | *0.82* |
| Per 10 g | *-1.45 (-4.03, 1.12)* | | | *1.21 (-1.38, 3.79)* | *0.37 (-1.75, 2.48)* | | *-6.8 (-18.5, 5.0)* |

*^a^ Values are based on multivariable linear regression models and reflect differences or percentage change (95%CI) in kidney outcomes for tertiles of animal or vegetable protein intake compared to the lowest tertile, and per 10 g of animal or vegetable protein intake per day.*

*^b^ Animal and vegetable protein intake are energy-adjusted using the nutrient residual method. Models are adjusted for child’s sex, age and body surface area at 6 y visit; for maternal age, educational level, and BMI at enrolment, for smoking and folic acid supplement use during pregnancy; for children’s ethnicity, gestational-age adjusted birth weight, for breastfeeding in the first four months of life, children’s television watching, total energy intake, energy-adjusted total fat intake, energy-adjusted sodium intake, and diet quality score (model 3 from main analysis). Models with animal protein intake are adjusted for vegetable protein intake and vice versa.*

*^c^ Albumin/creatinine ratio is log-transformed, therefore the regression coefficients reflect the percentage change rather than the absolute difference.*

*^d^ P_trend_ was obtained by including the number of the tertiles of protein intake as continuous variable in the model*

*Abbreviations: ACR, albumin/creatinine ratio; eGFR, estimated glomerular filtration rate.*

Supplementary Table S4. Associations of protein intake at the age of 1 y with kidney volume and function at 6 y in Dutch children only. ^a^

|  | **Kidney volume**  **(mm^3^)** | **eGFR_Creat_**  **(Schwartz 2009)**  **(ml/min per 1.73m²)** | **eGFR_CysC_**  **(Zappitelli 2006)**  **(ml/min per 1.73m²)** | **ACR**  **(% change)^c^** |
| --- | --- | --- | --- | --- |
|  | **n = 1,880** | **n = 1,385** | **n = 1,386** | **n = 1,960** |
| **Protein intake ^b^** |  |  |  |  |
| Tertile 1 | *Reference* | *Reference* | *Reference* | *Reference* |
| Tertile 2 | 1.69 (-0.56, 3.95) | 1.33 (-0.90, 3.56) | **2.12 (0.18, 4.07)** | -4.9 (-15.3, 5.5) |
| Tertile 3 | 0.36 (-3.18, 2.37) | 1.01 (-1.73, 3.76) | 2.15 (-0.25, 4.54) | -3.0 (-15.7, 9.7) |
| *P_trend_ ^d^* | *0.79* | *0.48* | *0.08* | *0.64* |
| Per 10 g | -0.96 (-2.62, 0.71) | 0.47 (-1.49, 1.15) | 1.27 (-0.14, 2.68) | -4.9 (-12.4, 2.5) |

*^a^ Values are based on multivariable linear regression models and reflect differences or percentage change (95%CI) in kidney outcomes for tertiles of protein intake compared to the lowest tertile, and per 10 g of protein intake per day. Bold numbers indicate statistically significant results (P<0.05)*

*^b^ Protein intake is energy-adjusted using the nutrient residual method.* *Models are adjusted for child’s sex, age and body surface area at 6 y visit; for maternal age, educational level, and BMI at enrolment, for smoking and folic acid supplement use during pregnancy; for children’s ethnicity, gestational-age adjusted birth weight, for breastfeeding in the first four months of life, children’s television watching, total energy intake, energy-adjusted total fat intake, energy-adjusted sodium intake, and diet quality score (model 3 from main analysis).*

*^c^ Albumin/creatinine ratio is log-transformed, therefore the regression coefficients reflect the percentage change rather than the absolute difference.*

*^d^ P_trend_ was obtained by including the number of the tertiles of protein intake as continuous variable in the model*

*Abbreviations: ACR, albumin/creatinine ratio; eGFR, estimated glomerular filtration rate.*

Supplementary Table S5. Associations of protein intake at 2 y with childhood kidney volume and function at 6 y. ^a^

|  | **Kidney volume**  **(mm^3^)** | **eGFR_Creat_**  **(Schwartz 2009)**  **(ml/min per 1.73m²)** | **eGFR_CysC_**  **(Zappitelli 2006)**  **(ml/min per 1.73m²)** | **ACR**  **(% change)^c^** |
| --- | --- | --- | --- | --- |
| **Protein intake** | **n = 660** | **n = 474** | **n = 474** | **n = 685** |
| **Model 1^b^** |  |  |  |  |
| Tertile 1 | *Reference* | *Reference* | *Reference* | *Reference* |
| Tertile 2 | 0.19 (-3.46, 3.84) | 1.33 (-2.14, 4.91) | -2.28 (-5.25, 0.68) | -15.4 (-31.7, 1.0) |
| Tertile 3 | 2.98 (-0.74, 6.70) | **4.03 (0.59, 7.47)** | 0.13 (-2.80, 3.07) | -14.5 (-31.0, 1.9) |
| *P_trend_ ^d^* | *0.12* | ***0.02*** | *0.95* | *0.08* |
| Per 10 g | **2.56 (0.19, 4.93)** | **3.21 (0.95, 5.47)** | 0.11 (-1.83, 2.05) | -9.4 (-20.0, 1.2) |
| **Model 2^b^** |  |  |  |  |
| Tertile 1 | *Reference* | *Reference* | *Reference* | *Reference* |
| Tertile 2 | -0.21 (-3.89, 3.47) | 1.20 (-2.31, 4.37) | -2.31 (-5.32, 0.70) | **-16.8 (-33.3, -0.4)** |
| Tertile 3 | 2.42 (-1.34, 6.17) | **2.28 (0.47, 7.43)** | -0.8 (-3.06, 2.91) | -15.8 (-32.4, 0.1) |
| *P_trend_ ^d^* | *0.22* | ***0.03*** | *0.94* | *0.06* |
| Per 10 g | 2.22 (-0.16, 4.61) | 3.06 (0.78, 5.35) | 0.00 (-1.97, 1.97) | -10.2 (-20.9, 0.4) |
| **Model 3^b^** |  |  |  |  |
| Tertile 1 | *Reference* | *Reference* | *Reference* | *Reference* |
| Tertile 2 | -1.30 (-4.34, 1.73) | -0.14 (-3.90, 3.63) | -2.27 (-6.50, 1.96) | -13.1 (-30.9, 4.7) |
| Tertile 3 | 0.98 (-1.83, 3.87) | 2.27 (-2.11, 6.66) | 1.60 (-5.88, 1.65) | -8.9 (-30.0, 12.2) |
| *P_trend_ ^d^* | *0.68* | *0.32* | *0.26* | *0.41* |
| Per 10 g | 0.21 (-3.23, 3.66) | 2.36 (-0.89, 5.61) | -0.90 (-3.70, 1.90) | -6.0 (-21.1, 9.1) |

*^a^ Values are based on multivariable linear regression models and reflect differences or percentage change (95%CI) in kidney outcomes for tertiles of protein intake compared to the lowest tertile, and per 10 g of protein intake per day. Bold numbers indicate statistically significant results (P<0.05)*

*^b^ Protein intake is energy-adjusted using the nutrient residual method.*

*Model 1 is adjusted for child’s sex, age and body surface area at 6 y visit.*

*Model 2 is additionally adjusted for and maternal age, educational level, and BMI at enrolment, for smoking and folic acid supplement use during pregnancy, and for children’s ethnicity, and gestational-age adjusted birth weight.*

*Model 3 is additionally adjusted for breastfeeding in the first four months of life, children’s television watching, total energy intake, energy-adjusted total fat intake, energy-adjusted sodium intake, and diet quality score.*

*^c^ Albumin/creatinine ratio is log-transformed, therefore the regression coefficients reflect the percentage change rather than the absolute difference.*

*^d^ P_trend_ was obtained by including the number of the tertiles of protein intake as continuous variable in the model*

*Abbreviations: ACR, albumin/creatinine ratio; eGFR, estimated glomerular filtration rate.*
